# The effect of forearm rotation on radiographic measurements of the wrist: an experimental study using radiostereometric analyses on cadavers

**DOI:** 10.1186/s41747-021-00209-1

**Published:** 2021-04-02

**Authors:** Janni Jensen, Hans B. Tromborg, Benjamin S. B. Rasmussen, Oke Gerke, Trine Torfing, Helle Precht, Ole Graumann

**Affiliations:** 1 Department of Radiology, Odense University Hospital, Sdr. Boulevard 29, 5000 Odense C, Denmark; 2 Research and Innovation Unit of Radiology, University of Southern Denmark, Odense, Denmark; 3 OPEN, Open Patient data Explorative Network, Odense University Hospital, Odense, Denmark; 4 Department of Orthopedic Surgery, Odense University Hospital, Sdr. Boulevard 29, 5000 Odense C, Denmark; 5 Department of Clinical Research, University of Southern Denmark, Odense, Denmark; 6 Department of Nuclear Medicine, Odense University Hospital, Sdr. Boulevard 29, 5000 Odense C, Denmark; 7 Research and Innovation Unit of Radiology, SDU, University of Southern Denmark, Odense, Denmark; 8 Health Sciences Research Centre, UCL University College, Niels Bohrs Alle 1, 5230 Odense, Denmark; 9 Department of Radiology, Lillebaelt Hospital, University Hospital of Southern Denmark, Kolding, Denmark

**Keywords:** Cadaver, Forearm, Radiography, Radiostereometric analysis, Wrist

## Abstract

**Background:**

Although dorsal/palmar tilt, radial inclination (RI), and ulnar variance (UV) are measurements commonly performed in wrist radiographs, the impact of forearm rotation on those measurements during the radiographic procedure is uncertain. Our aim was to determine the impact of supination and pronation on the reliability of measurements of tilt, RI, and UV.

**Methods:**

Tantalum markers were inserted into the distal radius of 21 unfractured cadaver forearms. The forearms were radiographed in different degrees of supination and pronation. The exact degree of rotation was calculated with radiostereometric analyses. Tilt, RI, and UV were measured by two independent readers in a random and anonymised fashion. Association between forearm rotation and radiographic measurements was examined using linear regression.

**Results:**

Forearm rotation significantly impacted the radiographically measured tilt. One degree of supination and pronation respectively increased and decreased palmar tilt with 0.68° and 0.44°, observers 1 and 2, respectively. As opposed to observer 1, observer 2 found that RI was significantly impacted by rotation with a slope of 0.08. Ulnar variance was not significantly impacted by rotation with linear regression slopes of 0.01° (95% confidence interval [CI] − 0.02–0.05, *p* = 0.490) and 0.02° (95% CI − 0.02–0.07; *p* = 0.288), observer 1 and observer 2, respectively.

**Conclusion:**

In unfractured forearms, the radiographically measured tilt was significantly affected by rotation. Palmar tilt increased with supination and decreased with pronation. Rotation significantly affected radial inclination, although of a magnitude that is probably not clinically relevant. No significant impact on UV was found.

## Key points

- The effect of forearm rotation on radiographic measurements of the wrist was investigated using radiostereometric analyses on cadaver forearms.
- According to both of the observers, forearm rotation significantly impacted reproducibility of the dorsal/palmar tilt, not significantly that of ulnar variance.
- Only one of two observers reported a significant impact of forearm rotation on reproducibility of the radial inclination but low variation could be clinically not relevant.
- Adhering to standardised radiographic positioning protocols is important to obtain reliability of radiological measurements of the wrist.

## Background

The distal radius is one of the most frequently fractured bones [1]. Treatment of a distal radius fracture is, at least partly, based on radiographic assessment. A number of radiographic measurements are commonly applied when quantifying and classifying a distal radius fracture [2, 3]. Following fracture reduction or surgery, the same measurements are used to describe the degree of anatomical alignment. Therefore, equally important to precise quantification of fracture displacement is accurate quantification of normal anatomy. Persistent mal-union of a distal radius fracture can potentially cause restricted range of motion and ultimately arthrosis [4, 5]. However, quantitatively describing the complex three-dimensional anatomy of the wrist from two-dimensional radiographs poses an uncertainty [6]. Furthermore, poor image and point of central ray may distort the landmarks if not centred directly on the region of interest [7]. Additionally, there may be differences in definition and understanding of the measurement method resulting in variation between different observers.

A standard radiographic wrist examination typically includes a posterior-anterior and a lateral view. The posterior-anterior view is usually taken with the palm towards the detector, the shoulder abducted 90°, and the elbow flexed approximately 90° and no ulnar or radial deviation of the wrist. The lateral view is taken with the ulna towards the detector, the elbow flexed approximately 90°, and no flexion, extension, or rotation of the wrist and forearm [8]. Forearm rotation during the radiographic procedure may impact subsequent radiographic measurements. Although methodologically not fully comparable, and with sample sizes ranging from 1 to 17, a small number of studies have previously demonstrated an association between forearm rotation and the radiographically measured values of tilt, radial inclination (RI), and ulnar variation (UV) [9–15].

Accurate quantification of forearm rotation is fundamental to accurate calculation of the impact of rotation. The abovementioned studies used goniometers to quantify rotation or went from full supination to neutral to full pronation. It has previously been suggested though that a goniometer may not accurately quantify forearm rotation [16]. Therefore, in the current study, the highly accurate radiostereometric analysis (RSA) was used to quantify supination and pronation with the radius as the centre of rotation. Radiostereometric analysis is a validated and highly precise research tool originally developed to measure the micro-motion of joint implants in relation to the surrounding bone [17, 18]. Traditionally, RSA is used to quantify motion between a rigid body (bone) relative to a potentially moving body (joint implant), *i.e*., relative motion. Absolute motion, on the other hand, is the quantification of change in positioning of a body part (*e.g*., rotation of the radius) between subsequent RSA exams. When using RSA, patient markers are inserted into cancellous and/or cortical bone which allows for a degree of rotation, for instance supination and pronation, to be calculated with the actual bone as the centre of rotation. This set-up, which adds precision to the calculation of the degree of rotation, has to the best of our knowledge not been applied previously.

The main objectives of this study were to investigate and quantify the influence of forearm rotation on the reproducibility of the radiographic measurements of tilt, RI, and UV.

## Methods

Twenty-one fresh frozen cadaveric human forearms severed mid-humerus were thawed and eligible for inclusion. The Regional Ethics Committee waived the requirement for official approval of this laboratory study according to Danish law of health §14 (Project-ID: S-20180077). All donor arms were fully anonymous and originated from individuals who had donated their bodies to scientific research.

### Eligibility

**Fig. 1:**
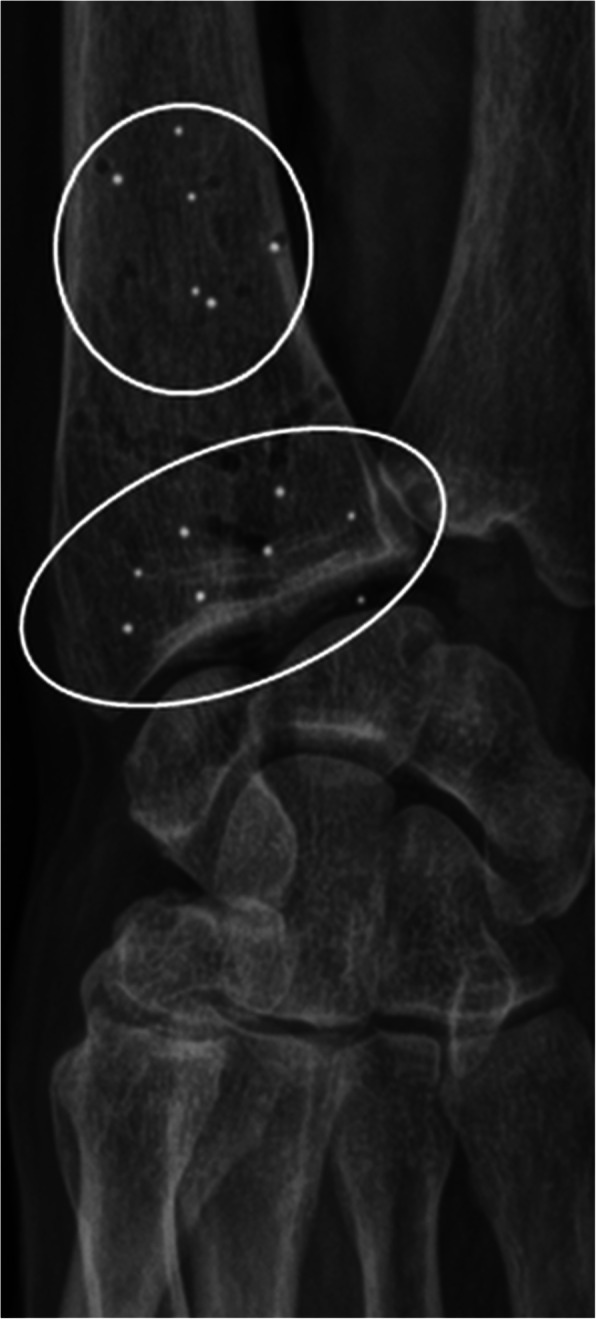
Cross-table posterior-anterior radiograph of a donor arm. Proximal and distal segments (white circles) with tantalum markers

Forearm rotation was quantified by RSA. Before RSA, exclusion criteria were donor arms with skeletally immature bones, congenital anomalies, or fracture sequela. After RSA, the exclusion was based on marker stability. Patient markers, spherical tantalum beads, sizes 0.8 and 1.0 mm, were inserted into the distal radius in two segments: 8–9 periarticular markers and 4–6 proximal markers (Fig. 1). The markers were placed in a circumferential manner. In the first six forearms, the markers were injected into the cancellous bone using a spring-loaded piston (RSA Biomedical AB, Umeaa, Sweden). In arms 7–21, the markers were placed in the cortical bone in pre-drilled holes and secured with bone wax.

Mean error (ME) of the rigid body is the mean difference between distances of markers between subsequent exams in millimeters. To avoid bias from movement of loose markers, donor arms with an ME > 0.35 mm were excluded [19]. Mean ME for proximal and distal segments respectively was 0.035 mm [range 0.006–0.284] and 0.048 mm [range 0.13–0.303]. All donor arms were included (11 right, 10 left).

### Imaging procedures

**Fig. 2:**
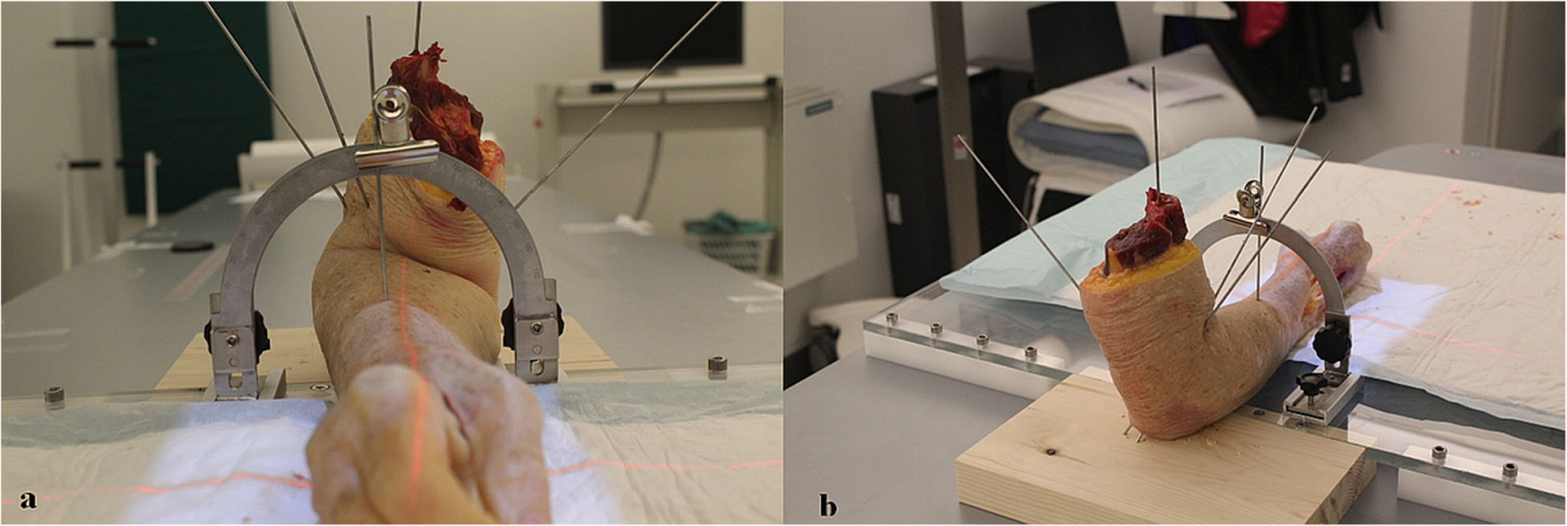
Entire cadaver forearm mounted on fixation platform with goniometer used for estimates of supination and pronation (**a**). Wooden board attached to the platform used for fixation of the forearm with K-wires through the humerus and ulna (**b**)

The donor arms were fixed to a custom-made radiolucent platform with the elbow flexed 90°. A Kirschner-wire (K-wire) drilled along the medullary canal of the humerus and 2–4 obliquely inserted K-wires through the olecranon secured the donor arms to the platform. This set-up allowed the radius to rotate over a stationary ulna. A K-wire in the proximal radius was used for estimates of supination and pronation against a goniometer (Fig. 2a, b). The 0° reference image was defined using the scaphoid-pisiform-capitate relationship, where the volar cortex of the pisiform was positioned in the middle third of the interval between the volar cortices of the scaphoid and the capitate [20]. From this baseline position, RSA images and radiographs were taken in various degrees of rotation. Using the goniometer, forearm rotations were made in steps of approximately 5° from the starting position and up to + 15° (pronation) and -15° (supination). Therefore, 3 supinated, a 0° rotated reference image, and 3 pronated images were taken of each forearm. RSA images and radiographs were taken in the same position, before rotating donor arms to the next position. With 7 sets of RSA and radiographic images per forearm and 21 forearms, a total of 147 RSA images and radiographs were analysed.

A mobile unit connected to the circuit of a ceiling-mounted tube enabled simultaneous exposure of RSA images (MiraMax1 and MultitomRax, Siemens Healtineers, Forchheim, Germany). X-ray tubes were positioned with a focus-to-detector distance of 140 cm and angulated 17° relative to the uniplanar calibration cage 43 (RSA Biomedical AB, Umeaa, Sweden). Double-exams were made of 14 reference images and the precision of RSA set-up assessed by comparing relative motion between double-exams. The ceiling-mounted tube was used for the acquisition of radiographs with the central ray directed at the radial styloid at a focus-to-detector distance of 100 cm. Posterior-anterior radiographs were taken cross-table. RSA radiographs were generated at 89 kVp and 14 mAs. Posterior-anterior and lateral radiographs were taken at 50 kVp and 2.5 mAs.

**Fig. 3:**
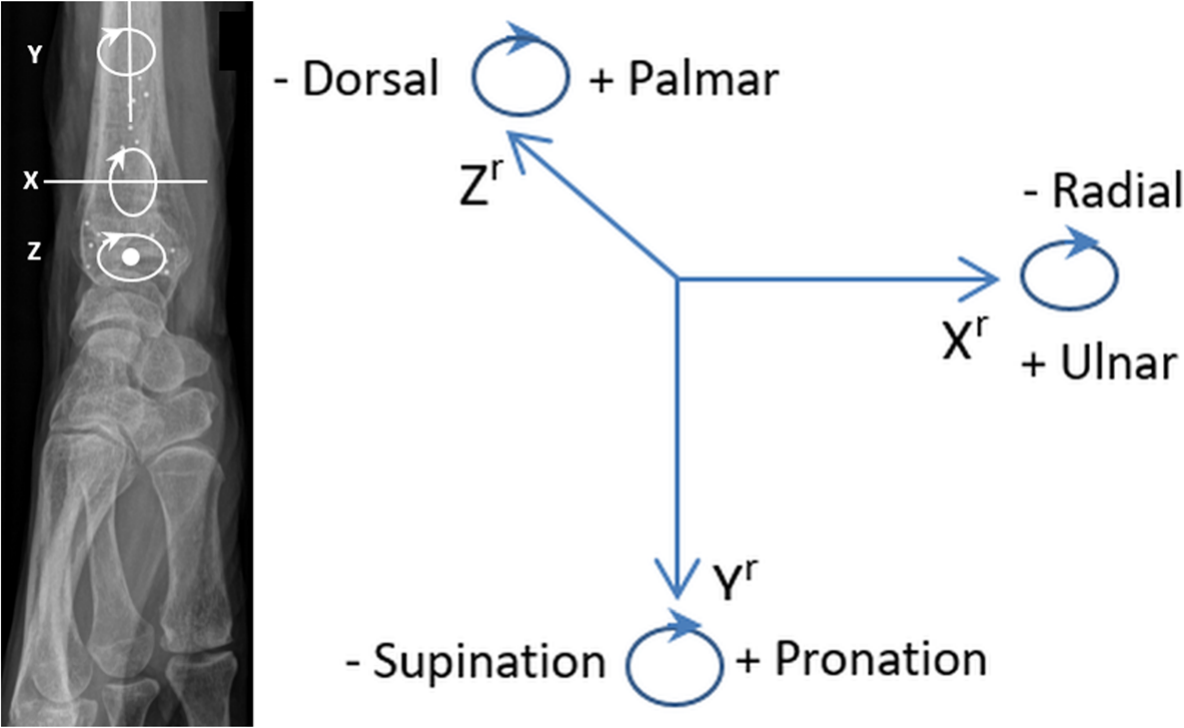
Rotation (*XYZ*^r^) in three dimensions according to the global coordinate system (right arm). Rotation around the *y*-axis (*Y*^r^) represents supination (−) and pronation (+)

The calibration cage contains known reference points at different levels. By taking bi-planer radiographs through the reference cage, positioning and movement of the inserted tantalum markers can be calculated in three dimensions using specialised software. RSA provides three-dimensional measurements of translation (*XYZ*^t^) and rotation (*XYZ*^r^). With the longitudinal axis of the radius positioned parallel to the *y*-axis of the calibration cage, rotation around the *y*-axis (*Y*^r^) was equivalent to supination (−) and pronation (+) in keeping with the RSA global coordinate system (Fig. 3). Signed values were reverted for left arms. Spatial marker distribution was assessed by the condition number (CN), calculated by the RSA software. Low CN’s indicate good spatial distribution of the markers where higher CN’s indicate close or collinear markers. RSA analyses were made by one author (J.J.) using the commercially available UmRSA software 7.0 (RSA Biomedical AB, Umeaa, Sweden).

**Fig. 4:**
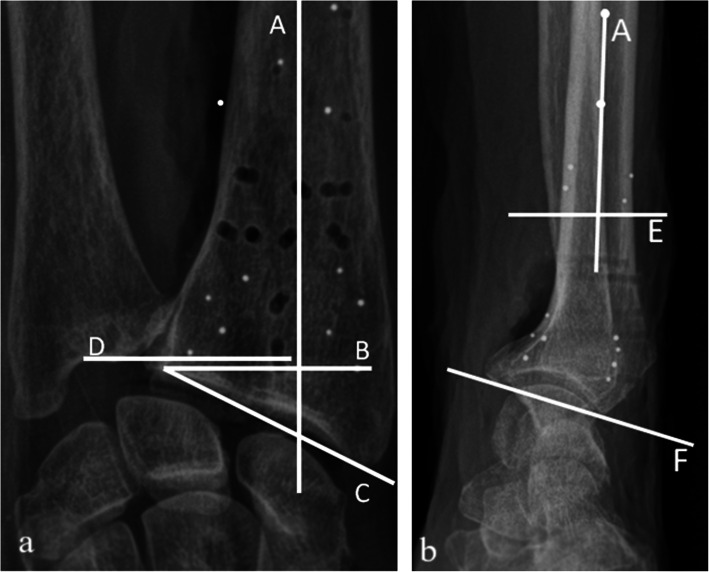
Radiographic measurements. Posterior-anterior projection (**a**); lateral projection (**b**). Line A represents the longitudinal axes of the radius (in both planes), determined by connecting two points (more than 2 cm apart) in the centre of the radial shaft. Line B is drawn from the most distal ulnar palmar corner of the radial articular surface and is perpendicular to line A. Line C connects the most distal ulnar palmar corner of the radial articular surface to the most distal part of the radial styloid tip. Line D abuts the most distal point of the articular surface of the ulna; it is parallel to line B thus perpendicular to the longitudinal axis of the radius. Line E is drawn at a convenient level perpendicular to the central long axis of the radius. Line F connects the most distal dorsal and palmar margins of the radial articular surface. The *ulnar variance* is defined as the length of the ulna relative to the radius, *i.e.*, the distance (mm) between lines D and B. The *radial inclination* is the inclination of the distal radial articular surface in the coronal plane, *i.e.*, the angle between lines B and C. *Tilt* describes the angulation of the distal radial articular surface in the sagittal plane, *i.e.*, the angle between lines E and F

The following radiographic measurements were made: tilt and RI to quantify the inclination of the distal radius articular surface in the sagittal and coronal planes, respectively, and UV to quantify the length of the ulna relative to the radius (Fig. 4). A musculoskeletal radiologist (T.T.) and a consultant hand surgeon (H.T.), with 25 and 19 years of experience, respectively, made all measurements independently in a randomised blinded fashion to the nearest 0.1 mm/degree. Measurements were made digitally in a Picture Archiving and Communication System (GE healthcare, IL, USA), collected and managed electronically using REDCap (Research Electronic Data Capture). To minimise systematic bias, observers had a tutorial on measuring techniques. Sixty-three images were double-reported by both observers. To minimise the risk of recall bias, there was a minimum of 4 weeks between the first and second reading.

### Statistical analyses

Mean CN and range were calculated. Standard deviations of differences between double-exams were calculated. A limit below which at least 95% of all measured differences between double-exams will fall was calculated as standard deviation × 2.160 (*t*-statistics, 95% = *n* − 1) [21]. Univariate linear regression models of tilt, RI, and UV on extent of rotation were applied. Goodness of fit was reported as *R*-squared values. Bland-Altman plots with limits of agreement illustrated inter- and intraobserver agreement [22, 23]. Assuming normality of differences, limits of agreement were estimates for the range that contains 95% of all population differences between or within the two observers. *p* values < 0.05 were considered statistically significant. Stata version 16 (StataCorp. 2019, TX) was used for statistical analyses.

## Results

Twenty-one unfractured donor arms (11 right, 10 left) were included and underwent RSA and radiographic imaging in various degrees of supination (-) and pronation (+) as calculated by RSA (range from -11.8° to 11.2°). The total number of images was 147. The goniometer measurements for supination (-) and pronation (+) were -15°, -10°, -5°, 0°, 5°, 10°, and 15°, thus overestimating the degree of rotation when compared to the RSA calculated values.

### Quality of set-up

The broader periarticular part of the distal radius allowed for better circumferential dispersion of markers. Probably, therefore, CN values were higher in the proximal segment than in the distal segment with mean values of 146 (range from 89 to 297) and 55 (range from 34 to 116), respectively. The precision of rotation, equivalent to supination and pronation (*Y*^r^), was 0.19° indicating that at least 95% of the differences measured between double-exams will be ≤ 0.19° (Table 1).

**Table 1:** Precision based on the 0° rotation double-exams (*n* = 14)

	***X***^**t**^ (mm)	***Y***^**t**^ (mm)	***Z***^**t**^ (mm)	***X***^**r**^ (°)	***Y***^**r**^ (°)	***Z***^**r**^ (°)
Standard deviation	0.03	0.01	0.04	0.13	0.09	0.13
Precision	0.06	0.02	0.09	0.28	0.19	0.28

*n* Number of double-exams, *XYZ*^*r*^ Rotation (degrees), *XYZ*^*t*^ Translation (mm). *XYZ* are axes in the three-dimensional coordinate system as depicted in Fig. 3. Precision = standard deviation × 0.975 *t* quantile

### Impact of rotation on radiographic measurements

The univariate regression model revealed a significant impact of rotation (RSA values) on the measured values of tilt for both observers (*p* < 0.001). Supination was found to increase, and pronation to decrease, the measurement of palmar tilt with 0.44 to 0.68° per 1° of rotation. Furthermore, measurements from observer 2 indicated a significant impact of rotation on RI with a slope of 0.08 (95% CI 0.02 to 0.13; *p* = 0.008). In contrast, no association between rotation and RI was found using the measurements from observer 1 (slope 0.05; 95% CI -0.02–0.11, *p* = 0.139). No impact of rotation on UV was evident for either of the observers (Table 2). The slopes for the impact of rotation on radiographic measurements using the goniometer values were (observer 1/observer 2) -0.44/-0.31 (tilt), 0.03/0.05 (RI), and 0.01/0.02 (UV).

**Table 2:** Mean measured values and results of univariate linear regression analyses for the impact of rotation on tilt, radial inclination, and ulnar variance using RSA values of rotation (*n* = 147)

		Mean ± SD (range)	Slope (95% CI)	***R***^**2**^	***p***-value
Tilt (dorsal/palmar)	O_1_	5.7 ± 9.1 (-22.0–21.3)	-0.68 (-0.87, -0.49)	0.25	< 0.001*
	O_2_	7.1 ± 6.9 -14.9–21.3)	-0.44 (-0.60, -0.29)	0.19	< 0.001*
Radial inclination	O_1_	26.1 ± 2.6 (18.6–33.0)	0.05 (-0.02, 0.11)	0.02	0.139
	O_2_	26.9 ± 2.4 (21.9–32.9)	0.08 (0.02, 0.13)	0.05	0.008*
Ulnar variance	O_1_	0.1 ± 1.5 (-3.7–4.6)	0.01 (-0.02, 0.05)	0.00	0.490
	O_2_	-0.2 ± 1.9 (-4.6–4.4)	0.02 (-0.02, 0.07)	0.01	0.288

*CI* Confidence interval, *n* Total number of images, *R*^*2*^ *R*-squared value, *SD* standard deviation, *O*_*1*_ Observer 1, *O*_*2*_ Observer 2. Tilt and radial inclination are reported in degrees (negative values for dorsal tilt, positive values for palmar tilt, ulnar variance in millimeters. *Statistical significance

### Agreement

**Fig. 5:**
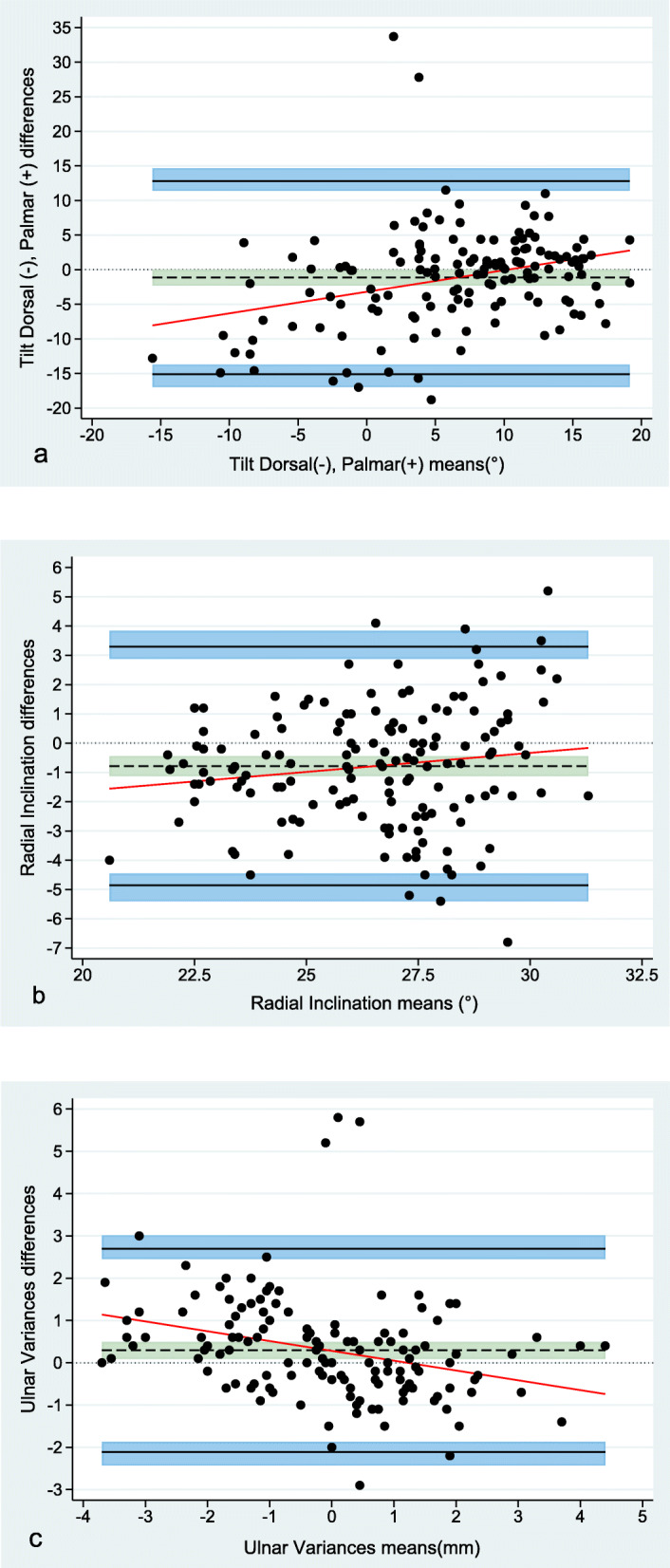
Bland-Altman plots displaying interobserver agreement (147 images). Differences between measurements are plotted against the mean of the measurements for tilt (**a**), radial inclination (**b**), and ulnar variance (**c**), respectively

**Fig. 6:**
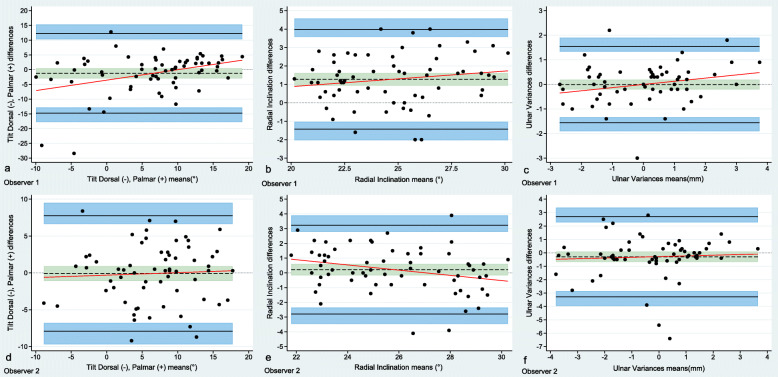
Bland-Altman plots displaying intraobserver agreement (63 images). Observer 1 (**a**–**c**) and observer 2 (**d**–**f**). Differences between 1st and 2nd measurement plotted against the mean of 1st and 2nd measurements

For measurements of tilt, RI, and UV, the mean measured difference, bias, between observers was -1.2°, -0.8°, and 0.3 mm, respectively. The Bland-Altman interobserver limits of agreement were -1.2 ± 13.9° for tilt, -0.8 ± 4.1° for RI, and 0.3 ± 2.4 mm for UV. The intraobserver mean measured differences were similar to the interobserver means. Table 3 summarises interobserver and intraobserver agreement. Bland-Altman plots with limits of agreement and 95% CI for interobserver and intraobserver agreement are presented to visually illustrate observer agreement (Figs. 5 and 6).

**Table 3:** Bland-Altman limits of agreement and bias (mean and SD). Interobserver agreement (*n* = 147), intraobserver agreement (*n* = 63)

		Bias, mean ± SD	Bias, 95% CI	Limits of agreement	Lower limit of agreement, 95% CI	Upper limit of agreement, 95% CI
Tilt	O_1 Intraobserver_	-1.25 ± 6.89	-2.99, 0.49	-14.76, 12.26	-17.79, -12.82	10.32, 15.29
	O_2 Intraobserver_	-0.83 ± 3.99	-1.09, 0.92	-7.92, 7.75	-9.68, -6.79	6.63, 9.51
	O_1&2 Interobserver_	-1.15 ± 7.11	-2.31, 0.01	-15.09, 12.80	-16.96, -13.70	11.40, 14.66
Radial inclination	O_1 Intraobserver_	1.27 ± 1.38	0.93, 1.62	-1.42, 3.97	-2.03, -1.04	3.58, 4.57
	O_2 Intraobserver_	0.22 ± 1.53	-0.17, 0.61	-2.78, 3.23	-3.46, -2.35	2.79, 3.90
	O_1&2 Interobserver_	-0.78 ± 2.08	-1.12, -0.44	-4.86, 3.29	-5.41, -4.45	2.89, 3.84
Ulnar variance	O_1 Intraobserver_	0.01 ± 0.79	-0.21, 0.20	-1.56, 1.55	-1.91, -1.34	1.33, 1.9
	O_2 Intraobserver_	-0.3 ± 1.53	0.09, -0.68	-3.29, 2.70	-3.96, -2.86	2.27, 3.37
	O_1&2 Interobserver_	0.29 ± 1.23	0.09, 0.49	-2.11, 2.69	-2.43, -1.87	2.45, 3.02

*CI* Confidence interval, *n* Number of images, *SD* Standard deviation, *O*_*1*_ Observer 1, *O*_*2*_ Observer 2. Tilt and radial inclination are reported in degrees (negative values for dorsal tilt, positive values for palmar tilt, ulnar variance in millimeters)

## Discussion

This study investigated the impact of forearm rotation on three radiographic wrist measurements, tilt, radial inclination, and ulnar variance. The results showed variation in measurements taken of the same arm in different positions.

Forearm rotation significantly affected tilt measurements, 1° of supination and pronation, respectively increased and decreased, palmar tilt with 0.44 to 0.68° (Table 2). Previous studies reported less pronounced magnitudes of impact, ranging from 0.32 to 0.47° [11, 13, 14]. This discrepancy may be explained by definition of a true lateral wrist radiograph. The study reporting an impact of 0.32° defined an image where the head of the ulna superimposes the distal radius as a true lateral [14]. Using the scaphoid-pisiform-capitate relationship, the head of the ulna usually superimposes *the dorsal* half of the radius; thus, their starting point differs from ours, potentially obscuring subsequent measurements [15]. Another explanation for differences in outcome could be the use of goniometers to measure rotation. Performing the regression analyses using goniometer values as opposed to RSA values decreased the impact of rotation from 0.44°/0.68° to 0.31°/0.44° in the current study. This is strikingly comparable to the abovementioned levels of impact from studies using goniometers to assess rotation. The goniometer measurements may be biased due to manual positioning of the K-wire against the goniometer. Additionally, the RSA-calculated rotation is based on circumferential markers placed directly on and within the radius.

Data from the current study did not support evidence of a clinically relevant impact of rotation on RI. Although data from observer 2 showed a significant impact of rotation on RI, the slope was only 0.08. Hence, approximately 13° of forearm rotation would change the measured value of RI with 1°. In line with this finding, DiBenedetto et al. [12] found that 10° of forearm rotation changed the measured value of RI with 1°. Conversely, Pennock et al. [11] reported an impact of 0.28 (slope) on RI. Differences in definition of landmarks may explain variations in outcomes. The aforementioned studies define angulation of the articular surface of the radius as a line connecting the radial styloid to the ulnar aspect of the articular surface/the sigmoid notch [11, 12]. In the present study, this landmark was defined as the most distal ulnar *palmar* corner of the radius “surface.” The point where the palmar aspect of the lunate fossa meets the sigmoid notch is usually seen as a demarcated sclerotic boarder, a remarkable constant finding [24]. Perhaps therefore, rotation has minimal impact on the radiographically measured value of RI in the current study.

Anatomically, the longitudinal axes of the radius and ulna are parallel in full supination. As the forearm rotates from supination to pronation, the radius gradually crosses over the ulna making it *appear* shorter relative to the ulna. Consequently, a change in UV is expected during rotation. Data from the current study did not support this presumption. Supination has been shown, though, to shorten the ulna relative to the radius when measured in 3 steps: full supination-neutral-full pronation [9, 10, 25]. Perhaps the lack of impact in the current study can be explained by the restricted rotation of approximately ±11°. Although methodologically different, using CT as opposed to radiographs, a non-correlation between forearm rotation and UV is supported in a recent study reporting a mean change in UV of 0.03 mm going from supination to pronation with a mean forearm rotation of 102° [26].

There are limitations in the current study. Data is based on rotation within a limited range of motion [11.2° to -11.8°]. Whether more extreme rotation would alter the results remains unknown. It is fair to assume though that a range of approximately ±11° represents the deviation most commonly seen in clinical practice. Furthermore, the clinical relevance of the widely spaced limits of agreement is not touched upon in this study, neither is accuracy of measurements. Prior to applying radiographic measurements in the treatment decision, it would be beneficial with more studies exploring the impact of the radiographic/radiologic procedures on the measured values. In this study, the impact of rotation on unfractured wrists is investigated. Another, equally important, question is if forearm rotation exhibits a similar impact on fractured wrists.

In conclusion, this study showed that the measured value of palmar tilt increased with supination and decreased with pronation of the unfractured forearm. Rotation had a significant effect on radial inclination, although of a magnitude that is probably not clinically relevant. No impact on the radiographic measurement of ulnar variance was established.

## Data Availability

Data are available based on reasonable request from the corresponding author.

## Abbreviations

CI: Confidence interval
CN: Condition number
RI: Radial inclination
RSA: Radiostereometric analysis
UV: Ulnar variation
